# Supplementary material for: Utilization of cardiovascular magnetic resonance (CMR) imaging for resumption of athletic activities following COVID-19 infection: an expert consensus document on behalf of the American Heart Association Council on Cardiovascular Radiology and Intervention (CVRI) Leadership and endorsed by the Society for Cardiovascular Magnetic Resonance (SCMR)
Source: J Cardiovasc Magn Reson. 2022 Dec 21;24:73. doi: 10.1186/s12968-022-00907-8 (PMC9767806; doi:10.1186/s12968-022-00907-8)
Supplement: Supplementary file 1 — Additional file 1. Supplemental Table 1. [file 12968_2022_907_MOESM1_ESM.pdf]

SUPPLEMENTAL MATERIAL

First Author (Year)	Pubmed PMID	N cases	Median or Mean Age	Post Covid Interval	CMR Tesla	LGE Pulse Seq.	LGE Analysis	Prev. of LGE [%]	Prev. of Ischemic Pattern LGE	Prev. of Non-Ischemic & Pericardial Pattern LGE	T1 Mapping Pulse Seq.	Location & native T1 Cutoff [ms]	Prev. of Abnormal Native T1 [%]	ECV Cutoff [%]	Prev. of Abnormal ECV [%]	T2 Pulse Seq.	Location & T2 Cutoff [ms]	Prev. of Abnormal T2 [%]
Breitbart[86]	34448040	56	Ulloa 45.7	71d	1.5T	PSIR GRE	ND	12%	~1%	11%	MOLLI	basal T1 > 1077; mid T1 > 1083; apical T1 > 1081	0.4% (of all evaluable segments)	ND	ND (mean ECV 27.5 ± 3.4 %)		basal T2 > 50; mid T2 >51; apical T2 > 53	2% (of all evaluable segments)
Bustin)[72]	34600236	20	46	54d	1.5T	high-resolution 3D IR-GRE	visual grading	65%	20%	45%	ND	ND	ND (T1 = 1028 ± 59 ms)	ND	ND (mean ECV 24.6 ± 2.5 %)	ND	ND	ND (T2 = 49±4 ms)
Cassar[93]	34693230	58	55	6m	3T	PSIR GRE	visual and quantitative (5SD)	23%	0%	23%	ShMOLLI 5(1)3(1)1	basal T1 > 1197; mid T1 > 1215; apical T1 > 1275	basal: 9%; mid: 2%; apical: 2%	basal: > 35%; mid > 36%; apical: > 38%	basal: 6%; mid: 0%; apical: 0%	T2-prep SSFP	basal: > 46; mid > 46; apical: > 56	basal: 2%; mid: 2%; apical: 4%
Clark[87]	34620179	50	27	71d	1.5T	PSIR GRE	visual	23%	0%	22.50%	MOLLI Pre: 5(3s)3; Post: 4(1)3(1)2)	97.5%% in controls: basal T1 = 999; mid T1 = 987; apical T1 = 988	ND	97.5%% in controls: basal = 25%; mid 23%; apical = 23%	NS	T2-prep SSFP	97.5%% in controls: 46 ms	NS
Galea[84]	34107985	27	54	20d	1.5T	IR TFE	visual and quantitative (5SD)	44%	33%	11%	MOLLI	T1>1027	41%	ECV>29.5%	43%	T2-prep SSFP	global T2>49.9	52%
Huang[48]	32763118	26	39	38 d	3T	ND	visual and quantitative (5SD)	58% with LGE and/or T2 abnormality	ND	31%	ND	ND	ND (median T1 = 1271 vs. 1237 ms)	ND (ECV = 28.2%)		T2-prep single-shot SSFP	ND (median T2 = 42.7 vs. 38.1	58% with LGE and/or T2 abnormality

SUPPLEMENTAL MATERIAL

First Author (Year)	Pubmed PMID	N cases	Median or Mean Age	Post Covid Interval	CMR Tesla	LGE Pulse Seq.	LGE Analysis	Prev. of LGE [%]	Prev. of Ischemic Pattern LGE	Prev. of Non-Ischemic & Pericardial Pattern LGE	T1 Mapping Pulse Seq.	Location & native T1 Cutoff [ms]	Prev. of Abnormal Native T1 [%]	ECV Cutoff [%]	Prev. of Abnormal ECV [%]	T2 Pulse Seq.	Location & T2 Cutoff [ms]	Prev. of Abnormal T2 [%]
Joy[75]	33975819	74	37	6m	1.5T		visual and quantitative (3SD)				MOLLI Pre: 5s[3s]; Post: 4s[1s]3s[1s]2s	septal/global T1 > 1072	7%	ND (ECV ~ 22%)	4%	ND	Septal/global T2 > 52.4	~8%
Knight[79]	32673505	29	64	37d	1.5T	ND	visual	~60%	ND	~50%	ND	ND	ND	ND	ND	ND	ND	ND
Kotecha[49]	33596594	148	64	68d	1.5T	PSIR FLASH	visual	49%	16%	10%	MOLLI 5s(3s)3s	septal ROI > 1076	~13%	ND	ND	T2-prep single-shot SSFP	septal ROI T2 > 52	~3%
Li[78]	33434112	40	54	158 d	3T	PSIR GRE	visual	1%	ND	ND	MOLLI Pre: 5(3)3; Post: 4(1)3(1)2	ND	ND	ND (ECV ~ 30%)	ND	ND	ND	ND
Luetkens[85]	33969316	8	8	8d	1.5T	ND	visual	38%	ND	ND	ND	ND	ND (T1 = 1044 ± 32 vs. 953 ± 32 ms; P<0.001)	ND	ND	ND	ND	13%
Ng[80]	33153536	16	68	56d	1.5T	ND	visual	25%	19%	6%	SMART1 (saturation-prepared)	global T1 > 1208	25%	ND	ND	ND	global T2 > 54.8	5%
Puntman[35]	32730619	100	49	56d	3T	ND	visual	32%		20%	MOLLI (scheme ND)	ND	73%	ND	ND	T2-FLASH	ND	60%

SUPPLEMENTAL MATERIAL

First Author (Year)	Pubmed PMID	N cases	Median or Mean Age	Post Covid Interval	CMR Tesla	LGE Pulse Seq.	LGE Analysis	Prev. of LGE [%]	Prev. of Ischemic Pattern LGE	Prev. of Non-Ischemic & Pericardial Pattern LGE	T1 Mapping Pulse Seq.	Location & native T1 Cutoff [ms]	Prev. of Abnormal Native T1 [%]	ECV Cutoff [%]	Prev. of Abnormal ECV [%]	T2 Pulse Seq.	Location & T2 Cutoff [ms]	Prev. of Abnormal T2 [%]
Raman[76]	33490928	58	55	2.3m	3T	PSIR FLASH	visual and quantitative (5SD)				shMOLLI	basal T1 > 1197; mid T1 > 1215; apical T1 > 1275	26%	ND (ECV ~ 30%)	ND	T2-prep single-shot SSFP	basal & mid LV T2 > 46; apical T2 > 51	2-6%
Urmeneta Ulloa[83]	34368419	57	59	81d	1.5T	ND	ND	26%	3.50%	22.80%	MOLLI 3(3)5 scheme	ND	ND (T1=996.4 ± 43.9 ms; n.s. difference with controls)	ND	19%	T2-prep TSE	ND (50.9 ± 4.3 vs. 48 ± 1.9; p<0.01)	ND
Wang[77]	33627143	44	48	103d	3T	PSIR TFE	visual and quantitative (FWHM)	29.50%	0%	29.50%	MOLLI (scheme ND; same for pre- and post-contrast)	ND	ND	ND	ND	ND	ND	ND
Webster[82]	34193197	17	14	72d	1.5T	ND	visual	ND	ND	ND	ND	ND	ND (T1= 973 ms; n.s. difference with controls)	ND	ND	ND	ND	ND
Wojtowic[81]	34708861	50	47	52d	1.5T	ND	visual	60%	ND	ND	ND	ND	2%	ND	ND	ND	ND	ND
Brito[51]	33223496	54	19	27d	1.5T	ND	visual	56%	ND	ND	ShMOLLI 5(1)3(1)1	T1 >= 990	19%	ND	ND	ND	T2 > 52	0%
Clark[54]	33332151	59	20	22d	1.5T	ND	visual	27%	ND	ND	ND	ND	ND	ND	ND (mean ECV 22.9 ± 2 %)	ND	ND	ND

SUPPLEMENTAL MATERIAL

First Author (Year)	Pubmed PMID	N cases	Median or Mean Age	Post Covid Interval	CMR Tesla	LGE Pulse Seq.	LGE Analysis	Prev. of LGE [%]	Prev. of Ischemic Pattern LGE	Prev. of Non-Ischemic & Pericardial Pattern LGE	T1 Mapping Pulse Seq.	Location & native T1 Cutoff [ms]	Prev. of Abnormal Native T1 [%]	ECV Cutoff [%]	Prev. of Abnormal ECV [%]	T2 Pulse Seq.	Location & T2 Cutoff [ms]	Prev. of Abnormal T2 [%]
Malek[57]	33474768	26	24	32d	1.5T	PSIR GRE	visual	4%	0%	4%	MOLLI	T1 > 1054	0%	> 31.9%	0%	T2-prep SSFP	> 50	4%
Rajpal[52]	32915194	26	19.5	23d	1.5T	ND	visual	46%	0%	46%	ND	T1 > 999	31%	29%	4%	ND	Max. segmental T2 >= 53	50%
Starekov[53]	33443537	145	20	15d	1.5 & 3T	PSIR GRE	visual	29%		28%	MOLLI	ND	ND (1.5T: 978 ± 40; 3T: 1128 ± 84 ms)	ND	ND	ND	ND	ND (1.5T: 48 ± 4; 3T: 49 ± 5 ms)
Szábo [61]	34848398	147	23	32d	1.5T	IR TFE	visual	2%	0%	2%	MOLLI 5(3)3 for pre and post	ND	ND (T1=958 ms)	ND	ND	T2-prep SSFP	ND (T2=45; n.s. difference vs. controls)	ND

Table S1.

Prev. = prevalence
Pulse Seq. = pulse sequence
ND = Not Defined
NS = Not specified
Grey shading = Athletic Cohorts
